# Medication Non-Adherence in Rheumatology, Oncology and Cardiology: A Review of the Literature of Risk Factors and Potential Interventions

**DOI:** 10.3390/ijerph191912036

**Published:** 2022-09-23

**Authors:** Vicente F. Gil-Guillen, Alejandro Balsa, Beatriz Bernárdez, Carmen Valdés y Llorca, Emilio Márquez-Contreras, Juan de la Haba-Rodríguez, Jose M. Castellano, Jesús Gómez-Martínez

**Affiliations:** 1 Department of Clinical Medicine, Miguel Hernandez University, 03550 San Juan, Spain; 2 Research Unit, Hospital General Universitario de Elda, 30600 Elda, Spain; 3 Rheumatology Department, La Paz University Hospital, 28046 Madrid, Spain; 4 Institute for Health Research Hospital La Paz (IdiPaz), 28046 Madrid, Spain; 5 Department of Oncologic Pharmacy, Santiago de Compostela University Hospital, 15706 Santiago de Compostela, Spain; 6 Medicine Department, Santiago de Compostela University, 15706 Santiago de Compostela, Spain; 7 Pharmacology Group, Health Research Institute of Santiago de Compostela (IDIS), 15782 Santiago de Compostela, Spain; 8 Adherence Group of the Sociedad Española de Farmacia Hospitalaria (ADHEFAR-SEFH), 28001 Madrid, Spain; 9 Fuencarral Health Center, 28034 Madrid, Spain; 10 Observatorio de Adherencia al Tratamiento (OAT), 28231 Madrid, Spain; 11 Treatment Adherence Chair, San Juan de Alicante University, 03550 Alicante, Spain; 12 Molino de la Vega Health Center, 21002 Huelva, Spain; 13 The Maimonides Institute for Biomedical Research of Cordoba (IMIBIC), 14004 Cordoba, Spain; 14 Centro Nacional de Investigaciones Cardiovasculares (CNIC), 28029 Madrid, Spain; 15 Centro Integral de Enfermedades Cardiovasculares, Monteprincipe University Hospital, 28660 Madrid, Spain; 16 Communiy Pharmacist, 08013 Barcelona, Spain

**Keywords:** adherence, medication, treatment, compliance, rheumatology, oncology, cardiology

## Abstract

Medication adherence is directly associated with health outcomes. Adherence has been reviewed extensively; however, most studies provide a narrow scope of the problem, covering a specific disease or treatment. This project’s objective was to identify risk factors for non-adherence in the fields of rheumatology, oncology, and cardiology as well as potential interventions to improve adherence and their association with the risk factors. The project was developed in three phases and carried out by a Steering Committee made up of experts from the fields of rheumatology, oncology, cardiology, general medicine, and hospital and community pharmacy. In phase 1, a bibliographic review was performed, and the articles/reviews were classified according to the authors’ level of confidence in the results and their clinical relevance. In phase 2, 20 risk factors for non-adherence were identified from these articles/reviews and agreed upon in Steering Committee meetings. In phase 3, potential interventions for improving adherence were also identified and agreed upon. The results obtained show that adherence is a dynamic concept that can change throughout the course of the disease, the treatments, and other factors. Educational interventions are the most studied ones and have the highest level of confidence in the authors’ opinion. Information and education are essential to improve adherence in all patients.

## 1. Introduction

Medication adherence can be defined as “the extent to which a person’s behavior—taking medication, following a diet, and/or executing lifestyle changes, corresponds with agreed recommendations from a healthcare provider” [1]. Improving medication adherence may have a greater impact on patients’ health than discovering any breakthrough therapy [2] and is therefore highly associated with health outcomes [1,3,4]. Furthermore, poor, or non-existent adherence has been shown to result in a substantial increase in mortality, morbidity, and healthcare costs [1,5,6,7,8,9,10,11,12,13]. While effective medicines are available for many conditions, it has been estimated that in most clinical scenarios, 50% of patients are non-adherent to their treatment [14]. This problem takes on a greater dimension in the case of chronic conditions, where long-term therapies are needed, and where full adherence is therefore less likely [15,16]. Adherence is a complex issue that can be influenced by various factors, which stem from more than the patient’s direct responsibility, as is commonly believed [1]. Although some of these factors are associated with intentional non-adherence (a conscious decision not to take the medication, e.g., because of fear of the drug’s adverse effects), most are linked to non-intentional non-adherence [1,17]. The reasons for non-adherence are diverse. The World Health Organization has proposed a classification based on their origin [1]: socioeconomic, healthcare system, patient, condition, and therapy. Adherence-enhancing interventions attempt to avoid negative effects on both patient outcomes and healthcare costs [18]. Given the multifaceted origin of non-adherence, numerous interventions for improving adherence have been proposed, ranging in complexity, scalability, technological base, organizational needs, etc. [19].

Adherence is a problem that has been extensively reviewed in the literature. However, most reviews have a narrow focus, covering only a specific disease or treatment. A comprehensive review of clinical fields is lacking, which would prove useful for specialists to expand their scope of the problem. The clinical reality is that clinicians have to manage a wide variety of patients with different clinical realities, so a review approach focused on their specific therapeutic field as a whole may be more educational and practical. Three important areas (rheumatology, oncology, and cardiology) were considered for this study, whose risk factors for non-adherence and possible interventions to improve adherence could be extrapolated to other pathologies. Although there are broad reviews on the topic of rheumatological medication adherence [20,21,22], some issues associated with this medical specialty are still not covered. In the area of oncology, there are several reviews, but mainly focusing on specific treatments [23] and/or diseases [24], lacking a synthesis that could help oncologists to address adherence problems in a more general way. In the case of cardiology, the reviews are focused mainly on certain diseases such as heart failure [25] or hypertension [26]. These three medical fields include a large number of chronic patients, and several of their pathologies are currently the leading causes of death [27]; therefore, a review of adherence covering them could help in the clinical management of a significant group of patients.

The main objectives of this project are (a) to identify factors that influence adherence, and (b) to propose potential interventions to improve levels of adherence, based on a bibliographic review and experts’ experience, with particular emphasis on the problem of adherence in rheumatology, oncology, and cardiology. The aim was to identify adherence patterns, both common and specific to the three areas, and then to associate them with specific interventions. Given the considerable amount of literature in the field, this review aims to provide a current and compact overview of this topic with the purpose of helping clinicians, especially rheumatologists, oncologists, and cardiologists to improve their patient management on adherence and, therefore, their health outcomes.

## 2. Materials and Methods

**Figure 1:**
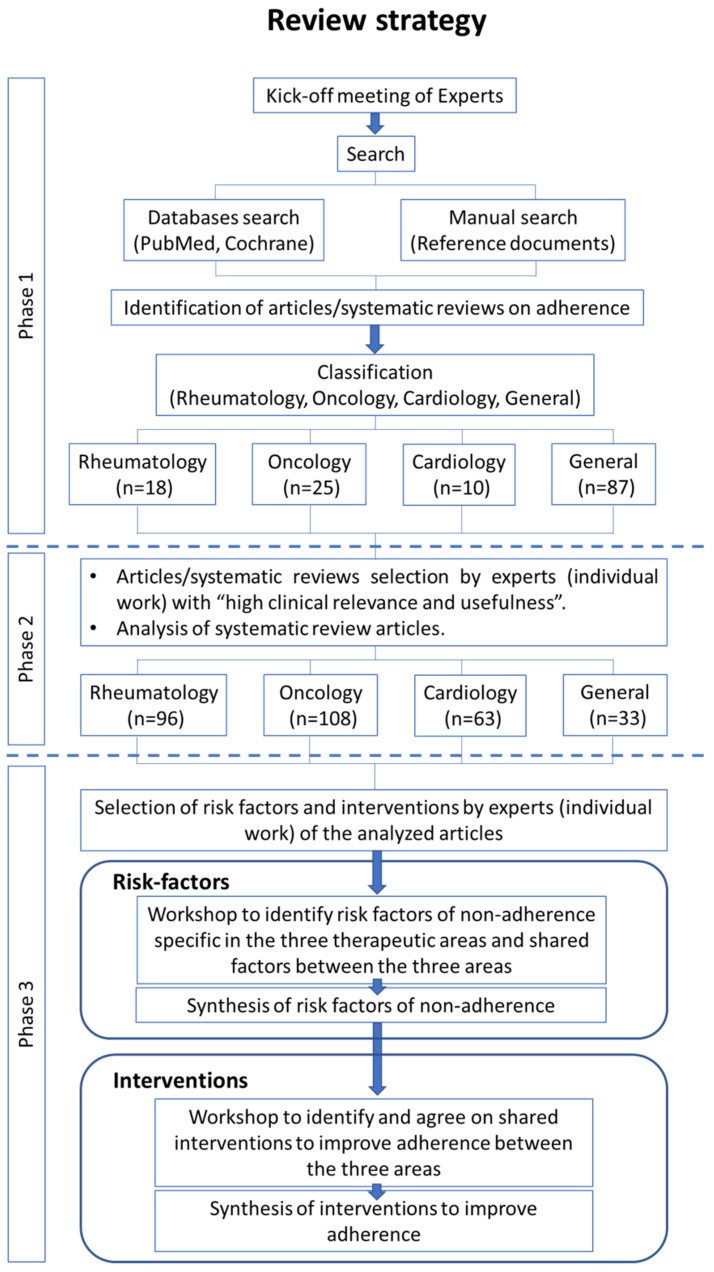
Study selection flow chart.

The project was carried out by a Steering Committee consisting of 1 rheumatologist, 1 oncologist (1 expert), 1 cardiologist, 3 general practitioners, 1 hospital pharmacist, and 1 community pharmacist. The members of the Steering Committee have previous experience performing literature reviews and are experts in adherence issues, being part of working groups in scientific societies on this subject. It was carried out in three phases (Figure 1). Phase 1 included a thorough search of the literature based on keywords selected by the Steering Committee (treatment adherence, compliance, treatment-adherence therapy, reinforcement of adherence to treatment regimen, monitoring adherence to medication regimen, clinical observation regimen, encouragement of compliance, assessment of compliance with medication regimen, adherence to medication regimen, compliance with treatment, compliance care assessment, and compliance care education). This search was conducted between November and December 2020 using PubMed and Cochrane databases, selecting articles/reviews published since 2014, according to the criteria agreed on with the experts. Furthermore, a bibliographic review of reference documents on adherence from different societies/organizations (Spanish Society of Hospital Pharmacy, World Health Organization, NICE, Farmaindustria) was carried out. All duplicate references were removed, and only articles/reviews addressing interventions or risk factors for treatment non-adherence in chronic diseases showing moderate to high certainty of the evidence were considered. The articles/reviews were classified into four areas of interest, depending on the subject or pathology: rheumatology, oncology, cardiology, and general. Phase 2 was conducted in January 2021. During this phase, experts individually reviewed the articles/reviews of their clinical specialty.

One rheumatology clinician reviewed papers classified within the rheumatology area, two oncology experts (a clinician and a hospital pharmacist) reviewed literature classified as oncology, one cardiology clinician reviewed studies classified as cardiology, and general medicine clinicians and community pharmacists (four experts) reviewed articles classified as general. The experts marked the articles/reviews following a color code based on the confidence on the relationship between specific risk factors or specific interventions and medication adherence or non-adherence: green when the authors have confidence, orange when the authors have doubts, and red when the authors have no confidence. In addition, the experts evaluated the usefulness for the clinical practice of each article/review marked as green. The experts exposed the results in a workshop, and reached an agreement on the articles and reviews to be selected for the project after a voting process of the Steering Committee. Phase 3 was carried out between February and March 2021. In this phase, the selected articles were analyzed, including those from the systematic reviews provided they were published after the year 2000.

Risk factors for non-adherence were extracted and classified within the five established dimensions by the World Health Organization [1]: socioeconomic, healthcare system, patient, condition, and therapy. A synthesis of the selected risk factors was made, and the factors shared between different therapeutic areas were identified in a workshop with the Steering Committee.

Interventions to enhance adherence were extracted and classified into five dimensions: educational, behavioral, cognitive, affective, and multifaceted. An exercise of synthesis was made in addition to an analysis to relate specific interventions to specific risk factors. These results were shared and discussed in a final workshop with the Steering Committee. Every risk and intervention on non-adherence previously classified was exposed and then discussed by the members of the Steering Committee. The notes from the debate served as the foundation for the current text.

## 3. Results

### 3.1. General Description of the Search Results

Figure 1 shows the results obtained throughout the course of the project. A total of 439 articles/reviews were found in databases. Once the selection and classification criteria were applied, the experts selected a total of 140 articles/reviews at the end of phase 1 (rheumatology: 18; oncology: 25; cardiology: 10; and general: 87). Considering the articles that met the selection criteria and those articles from systematic reviews addressing adherence in the selected groups of areas after the year 2000, in phase 2 a total of 300 articles were included (rheumatology: 96; oncology: 108; cardiology: 63; and general medicine: 33). Analysis of these publications yielded a total of 426 non-adherence-related factors, 60 belonging to the rheumatology area, 160 to oncology, 51 to cardiology, and 155 to the general area. Finally, after they were summarized and classified according to their clinical specialty, a total of 62 non-adherence-related factors were established, 11 of which were classified in the rheumatology area, 17 in oncology, 14 in cardiology, and 20 in the general area. Regarding interventions, a total of 30 were drawn from analysis of the selected articles.

### 3.2. Risk-Factors for Non-Adherence in Rheumatic Diseases

A summary of the risk factors associated with poor adherence in the rheumatology field is shown in Table 1.

Various socioeconomic risk factors were associated with poor adherence, such as the higher cost of medication [28], which depends on the coverage or non-coverage of the cost of medication by each particular healthcare system [21,29], and the fact of living alone, which is associated with a lack of social support, depression, and loneliness.

**Table 1:** Rheumatology risk factors of non-adherence.

Rheumatology	
**Socioeconomic**	High cost of medication [28] Living alone [30,31]
**Healthcare** **system**	Poor patient–healthcare provider relationship [21,32] Short duration of care time per patient Infrequent and low-intensity patient follow-up
**Patient**	Mental disorders [33,34,35,36] Lack of information and understanding about the pathology and treatment [37,38,39] Negative biases about treatment [28,40] Unhealthy lifestyle habits (smoking) [39,41,42]
**Condition**	Low disease activity [28,31,34,36,41,43,44,45,46,47,48,49,50] Mild pain [31,33,40,45,50]
**Therapy**	Adverse effects [40,47,51,52,53]

When studying the relationship between adherence and the healthcare system, the literature review showed that a poor patient–healthcare professional relationship, measured through questionnaires, is associated with treatment non-adherence [21,32]. This factor is highly complex since it includes several concepts, such as low confidence in the healthcare professional, a lack of patient participation in the decision-making process, poor satisfaction with the consultation, and a lack of communication patient–professional [32]. Other factors related to the healthcare system were agreed upon by experts: short duration of care time per patient, and infrequent and low-intensity patient follow-up.

Regarding rheumatology patients’ characteristics and their relationship with the lack of adherence, the literature review showed that suffering from different mental disorders such as depression, anxiety, or stress is associated with poor adherence, regardless of the type of rheumatological disease [33,34,35,36]. Additionally, the lack of information and understanding of the disease and treatment is also linked to a lack of adherence, since patients with a greater awareness of the pathology were shown to be more adherent to their prescribed treatment [37,38,39]. Furthermore, negative prejudice toward a given treatment was also linked to a lack of adherence [28,40], which indicates that the rheumatic patient’s disposition could be an important issue. Finally, regarding patients’ characteristics, unhealthy lifestyle habits, in particular smoking, are associated with less treatment adherence in rheumatological patients [39,41,42]. This fact was found in various rheumatic diseases, including psoriatic arthritis, rheumatoid arthritis, and ankylosing spondylitis [39,41,42], which pointed out the fact that adherence is merely a behavior that belongs to a cluster of healthy (including appropriate adherence) or unhealthy habits.

In the category of disease-related factors, a low disease activity was shown to increase the risk of non-adherence. Several parameters were included within disease activity, such as the presence of C-reactive protein, rheumatoid factor, serological activity, erythrocyte sedimentation rate (ESR), absence of affected joints, peripheral arthritis, DAS28 value, Ritchie joint index, visual analog pain score, duration of morning stiffness, grip strength, hemoglobin concentration, and Mallya and Mace classification [28,31,34,36,41,43,44,45,46,47,48,49,50]. Furthermore, this relationship between disease activity and non-adherence does not appear to be linked to any specific rheumatic disease. Moreover, we found that in several articles, pain was studied as a factor of non-adherence, finding that mild pain was linked to non-adherence in patients with rheumatic diseases [31,33,40,45,50]. In this case, it should be noted that most studies were carried out on patients with rheumatoid arthritis.

Regarding therapy-related factors in rheumatology, adverse effects from treatment were the only ones found to be linked to the risk of non-adherence [40,47,51,52,53]. This factor has a wide-ranging impact on adherence in rheumatology: several studies report its effects on different rheumatic diseases, including chronic arthritis, rheumatoid arthritis, systemic lupus erythematosus, and psoriatic arthritis [40,47,51,52,53].

### 3.3. Risk Factors for Non-Adherence in Oncology

Oncology is a very broad medical specialty, grouping together multiple diseases, syndromes, prognoses, and multiple treatment modalities. Perhaps, for this reason, a large number of non-adherence risk factors were found in the literature review (Table 2).

Socioeconomic factors linked to poor adherence have been found. For instance, in two breast cancer studies (one with European patients and another with American patients), single women with breast cancer were shown to have lower adherence to adjuvant endocrine therapy than non-single women [54,55]. A lack of social support was also shown to have an impact on treatment adherence [56,57,58,59,60,61,62]. Another socioeconomic-related risk for non-adherence was the fact of living alone [63,64]. The high cost of cancer medication was shown to strongly correlate with treatment non-adherence [65,66] according to various studies carried out in the US, given the poor coverage of the cost of medication by the public healthcare system.

Regarding healthcare system-related factors in the oncology field, a direct association between a poor patient–healthcare provider relationship to a lack of treatment adherence was shown [67,68]. Moreover, non-adherence was associated with short follow-up visits for cancer patients [56,69,70]. Supply problems of community pharmacies were also shown to be related to poor adherence [63]. Finally, two studies linked the high costs of hospital care with treatment non-adherence [71,72].

Regarding cancer patients’ characteristics and their relationship with the lack of adherence, many articles were found to study this dimension. Several studies have found a link between young patients (aged 15–30 years old), and lower medication adherence [54,73,74,75,76]. Another factor, linked to the patient’s biology and patient’s age, is the desire or intention to become pregnant. Wanting to become pregnant has been linked to early discontinuation/lack of adherence in patients with chronic myeloid leukemia [62]. In this case, fear of medication’s adverse effects on the fetus or the likelihood of becoming pregnant were included as potential explanations for early discontinuation. Given that these studies included small sample sizes, the experts in oncology decided to exclude them from the final selection of factors, as these may not be representative or generalizable. The presence of mental health problems (including depression, anxiety, stress, previous antidepressant use, cognitive symptoms, and cancer-specific psychological distress) has been reported as a key factor in determining a patient’s adherent behavior [56,58,60,69,70,75,77,78,79,80,81,82,83,84]. This factor was shown to be independent of the cancer type or other patients’ characteristics. The patient’s prejudices and attitudes are also important. Examples of the influence of negative biases on oncology patients towards specific treatments have been identified in many articles/reviews as central factors impacting cancer treatment adherence. These biases include negative beliefs about medication, negative emotions and feelings, fear of adverse effects, a low perceived need by the patient to take the medication, concerns around the medication, and low patient satisfaction with the medication [56,60,62,67,68,69,77,85,86,87,88]. Within the same dimension, patients’ perceived lack of self-efficacy, in terms of taking the medication, has been shown to be correlated with a lack of treatment adherence [89,90,91]. Another factor that has been shown to determine non-adherence is the difficulty in symptom control, and in some cases, the frustration that comes with it [92]. The most important unintentional factor related to non-adherence is forgetfulness, which has been shown to be responsible for low adherence in various types of cancer [56,62,65,66,69,70,86,93,94,95,96,97,98]. Adherence in itself is a habit, and various unhealthy habits, in particular, smoking and alcohol consumption, correlate with a lack of adherence [56,62,85,99]. In terms of the patient dimension, studies have shown a link between low adherence and health literacy as explained by the difficulty in understanding the drug’s instructions or the label [66,97]. Taking this information into account, the oncology experts identified and agreed that the main and most important non-adherence factors in cancer patients within the patient’s characteristics dimension are patients’ young age, mental disorders, negative biases regarding the treatment, lack of information and understanding about the disease and treatment, negative biases about prognosis, frustration/difficulty of symptoms control, forgetfulness, and unhealthy lifestyle habits.

Condition-related factors are another dimension that includes barriers to adherence. Several studies have shown that the presence of comorbidities can predict low adherence rates [54,56,60,69,70,72,74,75,76,79,83,84,90,100,101,102,103,104,105,106,107,108,109]. The type of treatment in oncological patients has also been shown to impact adherence. Interestingly, we found that patients with breast cancer who underwent a mastectomy showed lower treatment adherence compared to patients who underwent conservation surgery [56,61,69,76,79,103,110,111]. It should be noted that, in this context, experts considered that the type of surgery may be masking patients’ perception of the disease’s severity. Yet another factor specifically in breast cancer is the type of carcinoma: patients with ductal carcinoma in situ are less adherent to adjuvant endocrine therapy than those with dissemination of the disease [58]. However, it should be noted that in this case, this phenomenon appears to be linked to patients’ perception of therapy’s risks and benefits [58].

Therapy-related barriers to adherence in oncology have also been extensively studied in the literature. There is extensive evidence linking the incidence of adverse effects to poor therapeutic adherence [56,62,70,80,91,94,96,98,105,106,112,113,114,115,116,117]. Adverse effects may be both physical (musculoskeletal and joint pain, the medication’s unpleasant taste) and psychological (cognitive symptoms). In this context, cumulative toxicity has been also demonstrated as a factor correlating with poor adherence. Probably related to this factor, treatment duration has been linked to a lack of adherence, regardless of the disease type (breast cancer, chronic myeloid leukemia, renal cell carcinoma, etc.), medication class, and the time considered for non-adherence (6 months, 18 months, etc.) [56,60,70,96,98,115,118,119]. For this reason, chronic patients and those with long-term cancer are especially vulnerable to low adherence.

Finally, another therapy-related factor is treatment complexity, which, in the context of oncology, is associated with advanced disease stages and is usually accompanied by a greater number of drugs taken. Treatment complexity also appears to intensify ‘depressive symptoms’, consequently having a negative impact on non-adherence [56,60,70,78,100,106]. Furthermore, it often introduces changes in the way cancer medication is taken, which has been shown to correlate with low adherence in these patients (such as variations when taking the medications in the morning vs. in the afternoon/evening, ingestion with or without food, etc.). In the dimension of therapy-related factors for non-adherence, experts agreed on the importance of adverse effects, the therapeutic regimen’s high complexity, and treatment duration as non-adherence factors in cancer patients.

### 3.4. Risk Factors for Non-Adherence in Cardiology

After the literature review, various factors for non-adherence in cardiology were identified (Table 3). To reach a consensus on these shared non-adherence risk factors, the experts considered that some of the factors identified in the literature, mainly those related to the costs of the treatment for the patient, are highly dependent on the healthcare system of some countries, but not so in others where the public system covers the cost of healthcare and medication.

Low educational levels have been shown to correlate with lower levels of adherence, according to studies carried out in patients with hypertension, acute myocardial infarction, and congestive heart disease [120,121,122]. It is important to highlight that the populations in which the data were gathered were from the US and Chile, so these data may not be extrapolated elsewhere. Another socioeconomic factor related to non-adherence in the cardiology area is the fact of living alone, according to experts’ experience.

Regarding the association between healthcare system factors and adherence in cardiac patients, the relationship between healthcare professionals and patients became evident in various studies. A poor relationship, noted in questionnaires assessing satisfaction in primary care centers, was linked to lower levels of treatment adherence [123]. In these questionnaires, different aspects of the patient–provider relationship were evaluated, which included the perception of the way a patient is welcomed into the office by the healthcare professional, the perception of their capacity, the perception of negative aspects in communication, the impossibility of contacting the prescribing healthcare professional, and a short consultation time [123]. Other non-adherence factors that the experts considered to be conditioned by and related to poor patient–health professional relationships include short duration of care time per patient and infrequent patient follow-up.

Regarding the association between patient-related factors and adherence in cardiac patients, one of the factors encountered was ethnicity. African Americans or non-Caucasians have shown lower levels of therapeutic adherence regardless of treatment type and cardiovascular disease [124,125]. Mental factors have also appeared in the literature review on this topic in the cardiology field. It has been shown in various studies on hypertensive patients that mental health status influences their treatment adherence [123,126,127,128,129]. Furthermore, in patients over 65 years of age treated with statins, dementia and depression constitute a key factor that reduces long-term adherence [127]. Similarly, stress has also been associated with lower levels of adherence to antihypertensive therapy [123,129]. Several studies concluded that forgetfulness is the main factor in non-adherence to blood pressure-lowering medications [130,131]. Other cardiac patient-related non-adherence factors that were agreed on and considered relevant by experts are unhealthy lifestyle habits and negative biases about treatment, especially in patients undergoing statin therapy.

**Table 3:** Cardiology risk factors of non-adherence.

Cardiology	
**Socioeconomic**	Living alone
**Healthcare** **system**	Bad patient–healthcare provider relationship [123] Short duration of care time per patient Infrequent and low-intensity patient follow-up
**Patient**	Mental health problems [123,126,127,128,129] Forgetfulness [130,131] Unhealthy lifestyle habits Negative biases about treatment
**Condition**	Low disease activity Mild/moderate pain
**Therapy**	Adverse effects [132] High complexity of the therapeutic regimen [126,133] Type of treatment [133,134,135]

In general, there is a lack of confidence in non-adherence factors related to the disease analyzed in the articles; nevertheless, the experts consider that there are two factors related to the lack of adherence in cardiac patients: low disease activity and mild/moderate pain.

Regarding the therapy dimension, a large number of adverse effects can lead to poor therapy adherence in cardiac patients [132]. Among these adverse effects, we found developing liver and kidney damage in the case of statins, and bleeding events in the case of anticoagulants [132]. Not only adverse effects are linked to poorer adherence, but also complex treatment regimens [126,133]. Both high doses and the use of multiple medications can lead to decreased treatment adherence in cardiac patients [123,133]. Another aspect that determines adherence in terms of the therapy dimension is the class of prescribed drugs (type of treatment). Patients treated with diuretics and beta-blockers generally show less adherence than those treated with calcium channel blockers or ACE inhibitors in hypertensive patients [133,134,135].

### 3.5. Shared Non-Adherence Risk-Factors

In addition to the articles/reviews analyzed in this project for three specific therapeutic areas, other articles that studied adherence in general, without mentioning any specific pathology, were also reviewed. Considering both, the general non-adherence risk factors and the specific risk factors for the three areas analyzed in this study, the experts reached a consensus on shared non-adherence risk factors (Table 4). These factors have been discussed in each of the pathologies described.

**Table 4:** Shared risk factors of non-adherence agreed by experts.

Socioeconomic	
**Socioeconomic**	Living alone
**Healthcare system**	Bad patient–healthcare provider relationship Short duration of care time per patient Infrequent and low-intensity patient follow-up
**Patient**	Young age Negative biases about treatment Mental disorders Unhealthy lifestyle habits Forgetfulness Negative biases about prognosis Frustration/difficulty of symptoms control Lack of information and understanding about the pathology and treatment
**Condition**	Low disease activity Mild/moderate pain Perception of gravity Comorbidities
**Therapy**	Adverse effects High complexity of the therapeutic regimen Type of treatment Long treatment duration

### 3.6. Interventions for Improving Adherence

Interventions for improving medication adherence can be divided into the following dimensions: educational, behavioral, cognitive, affective, and multifaceted. The list and classification of the interventions for therapeutic adherence improvement resulting from this study can be seen in Table 5.

Most of the interventions analyzed included an educational dimension, highlighting the importance of educating patients about their therapies, their appropriate administration, and the treatments’ risks and benefits [136,137,138,139,140,141,142,143]. Education should be given to both patients and healthcare professionals. For the information provided to the patient to be effective, training of healthcare professionals is needed to enhance their communication skills. Educational interventions were the most studied and have the highest level of confidence among all the interventions actions. Information and education are essential for improving adherence in all patients and should be carried out through the coordination of healthcare staff [137,138]. In the case of educational interventions, several examples were found, one of them being educational sessions [4,144,145]. The aim of these is to provide patient education on aspects of both their disease and treatment, as well as any relevant health-related information. An additional related type of educational intervention is assessing the awareness about the treatment through interviews, tests, or questionnaires [137,138]. This can be performed, for example, through an interview in which the patient comes with their medications and the healthcare professional asks them how they take each one. In this way, the patient’s awareness is assessed, and any problems are identified, with appropriate information then being provided. Another educational intervention that can be carried out is periodic follow-up visits, both face-to-face and via phone calls, to advise the patient on their pathology and treatment regimen [137,138].

In general, most of the behavioral, cognitive, and affective interventions reviewed for improving adherence have had a smaller effect than the educational interventions [136,137,138,139,140,141,142,143]. Interventions classified as behavioral include actions such as reminders to patients, which can be carried out by various means: email, refilling prescriptions, personal or automated phone calls, automated or mobile alarms for taking medication, etc. [19,145,146]. Another behavioral intervention found in the review and related to reminders is evaluating and monitoring adherence and the severity of symptoms through phone calls or using mobile or web applications [19,145,146]. Other strategies, in this case, treatment-related, may be classified in the behavioral category. These include simplifying and adjusting doses or therapeutic regimens [19,145,147], and several strategies for removing barriers relating to taking medication [4,144,145].

**Table 5:** Potential interventions to improve adherence.

Intervention Type	
**Educative**	High cost of medication [28] Living alone [30,31]
	Poor patient–healthcare provider relationship [21,32] Short duration of care time per patient Infrequent and low-intensity patient follow-up
	Mental disorders [33,34,35,36] Lack of information and understanding about the pathology and treatment [37,38,39] Negative biases about treatment [28,40] Unhealthy lifestyle habits (smoking) [39,41,42]
	Low disease activity [28,31,34,36,41,43,44,45,46,47,48,49,50] Mild pain [31,33,40,45,50]
	Adverse effects [40,47,51,52,53]
**Behavioral**	● Use of reminders [19,145,146]: ○ Email ○ Refilling prescriptions ○ Personal or automated phone calls ○ Automated or mobile alarms for taking medication ○ Electronic reminders individualized or mass mobile text messages ○ Mobile apps. For example, Medisafe and RememberMed ○ Use of pill boxes, automatic dispensers, electronic pill boxes with support audio-visual, pill containers with reminders or personalized ○ Dosage (by the healthcare professional) ○ Medication calendars, organizers, or diaries ○ Establish a routine that links taking medications with daily events or taking other medications ○ Involvement of family members/caregivers in making reminders ○ Put the medication in a visible place ● Evaluation and monitoring of adherence and severity of symptoms through phone calls or use of mobile or web applications [19,145,146]. ● Simplification/adjustment of doses or therapeutic regimens [19,145,146]: ○ Combination pills, fixed-dose combined polypill ○ Reduce the frequency of dosing, for example, with drugs with prolonged half-life or prolonged release ○ Change the medication formulation according to the individual characteristics of each patient ● Strategies for removing barriers related to taking medication [4,144,145]: ○ Improvements in the format and size of the instructions of the drug leaflets ○ Easy to use packaging ○ Medicine containers labeled with icons ○ Display of pictograms or dosage on the medicine box ○ Swallowing training
**Cognitive**	● Improvement of the patient–healthcare provider relationship [19,145,148,149,150]: ○ Provide the patient or family member/primary caregiver with communication channels with the healthcare professional (reactive contact) and provide the healthcare professional communication channels with the patient or family member/primary caregiver (contact proactive) ○ Training of healthcare professionals to improve their communication skills with patients ○ Skills-enhancing patient-centered care models that improve interpersonal skills ○ Routine reminders from professionals about the importance of adherence to increase patient satisfaction with their treatment ○ Open and cooperative communication between the different healthcare professionals and patients to improve the quality of care, patient satisfaction, and medication adherence ○ Understanding of the modifiable psychological factors of the patient by the healthcare professional ○ Individualized monitoring of beliefs related to treatment ● Encourage patient participation in decision making regarding the treatment [145,151]. ● Promote the creation of patient groups where experiences and healthy lifestyle habits to improve adherence can be shared [4]. ● Actions of Pharmaceutical Care Services [4]: ○ Pharmacotherapeutic follow-up and review of medication use ○ Review and conciliation of medications in polymedicated and multi-pathological patients (drug interactions, dosage schedules), consultations for the resolution of problems related to medication that result in negative results to the medication, development of care, and follow-up plans ○ Care plans ○ Telephone follow-up of medication ○ Structured advice and follow-up in the dispensing of a new medication or modification of one that is already being taken in the community pharmacy, ensuring that the patient knows why to take it, how much, how, and for how long, and monitoring of prescriptions not dispensed ● Visualization of the progress of the disease through a multidimensional questionnaire on health status and subsequent clinical evaluation [4]. ● Strategies that facilitate/improve patient care [4,151]: ○ Decrease in waiting times ○ Short intervals between appointments ○ Home visits ○ Collaborative care ○ Reduction in the frequency of visits ○ Liaison with the general practitioner ○ Testing at the point of care ○ Planning discharge and follow-up visits ○ Multidisciplinary coordination between healthcare professionals and organizational change to improve the continuity and efficiency of patient care
**Affective**	● Social support to the patient [19,145]: health tutor, group programs, psychological therapy, e.g., ● Personal or telephone semi-structured motivational interviews by the healthcare professional to encourage patient empowerment [145]. ● Effective management of mood-related problems and symptoms experienced before and during therapy such as the early identification of emotional distress and therapeutic interventions to improve psychological well-being [152]. ● Advice to the patient by healthcare professionals [4,19,145,153,154]: ○ Treatment: ▪ Benefits ▪ Importance ▪ Objective ▪ Action mode ▪ Causes of low effect ▪ Correct use of device medication ▪ Medication adherence ▪ Security ▪ Adverse events ○ Target disease, symptoms, and health ○ Lifestyle (diet, exercise, smoking) ○ Negative/incorrect beliefs about the disease medication ● Preparation of written material that addresses the benefits of maintaining positive behaviors to overcome obstacles that hinder adherence and telephone calls to reinforce these positive behaviors [155].
**Multifaceted**	Comprehensive Geriatric Assessment: Determination of the medical, psychosocial, functional, and environmental resources and problems of an elderly person, and creation of a general plan for treatment and follow-up [151]. Combination of educational and behavioral interventions. Combination of educational and cognitive behavioral interventions. Combination of educational and affective interventions. Combination of educational and cognitive behavioral interventions. Combination of educational, behavioral, cognitive behavioral, and affective interventions. Combination of behavioral, cognitive-behavioral, and affective interventions.

The cognitive aspect is very important in the adherence context; belonging to this category, several interventions were found through the literature review, the most important one being an improvement in the patient–healthcare provider relationship [19,145,148,149,150]. This is a complex problem, and one for which different actions may be taken, ranging from providing new communication channels to improving patients’ and healthcare providers’ communication skills [19,145,148,149,150]. Other ways explored for improving adherence through cognitive interventions include encouraging patient participation in treatment-related decision making [145,151], promoting the creation of patient groups where experiences and healthy lifestyle habits for improving adherence can be shared [4], diverse actions by pharmaceutical healthcare services [4] (pharmacotherapeutic follow-up and review of medication, care plans, telephone follow-up of medication, e.g.,), visualizing the disease’s progress through a multidimensional questionnaire on health status [4], and several strategies that facilitate and improve patient care [4,151] (decrease in waiting times, short intervals between appointments, home visits, e.g.,).

The literature review has also provided several interventions that may be classified inside the affective dimension, which include providing social support for the patient, which can be achieved through a health tutor’s participation, group programs, and even psychological therapy [19,145]. Another way of providing effective support for increasing patients’ treatment adherence is for healthcare professionals to carry out semi-structured in-person or telephone motivational interviews to encourage patient empowerment [145]. Effective management of mood-related problems and symptoms experienced before and during therapy such as early identification of emotional distress and therapeutic interventions for improving psychological well-being is another intervention that appeared in this literature review [152]. Lastly, in the affective dimension, we may mention other actions such as healthcare professionals’ advice for patients [4,19,145,153,154] (regarding treatment, target disease, symptoms and health, lifestyle, or beliefs about the disease medication) and emphasizing the benefits of a positive attitude, using written material or phone calls [155].

To address those cases combining several risk factors at the same time, many authors suggest combining several interventions through multifaceted programs. According to the World Health Organization [1], multifaceted—the use of two or more adherence-improving strategies—interventions should take into account “a combination of actively involving patients in their own healthcare decisions, provision of appropriate support, multidimensional educational programs that teach behavioral skills to the patient to enhance his or her adherence and tailoring of the regimen to fit the patient”.

### 3.7. Proposed Interventions to Address Non-Adherence Risk Factors

Improving adherence requires a sustained and dynamic process, in which different interventions should be considered, and the strategy to be followed should be personalized. Both intentional and unintended factors as well as the specific reasons that patients have for not taking the medication need to be targeted [18]. For this reason, most of the articles that address this issue propose conducting personalized interventions for each patient [136,137,138,139,140,141,142,143]. Greater satisfaction with the treatment was the strongest predictor of adherence, suggesting the need to improve the patient–healthcare professional relationship and implement care models focused on each particular patient in order to ensure optimal results [136,137,138,139,140,141,142,143].

For this approach, it is necessary to previously identify the reasons for non-adherence and adjust the intervention for each individual. An intervention that was effective at increasing medication adherence in one patient may not be effective in other.

**Figure 2:**
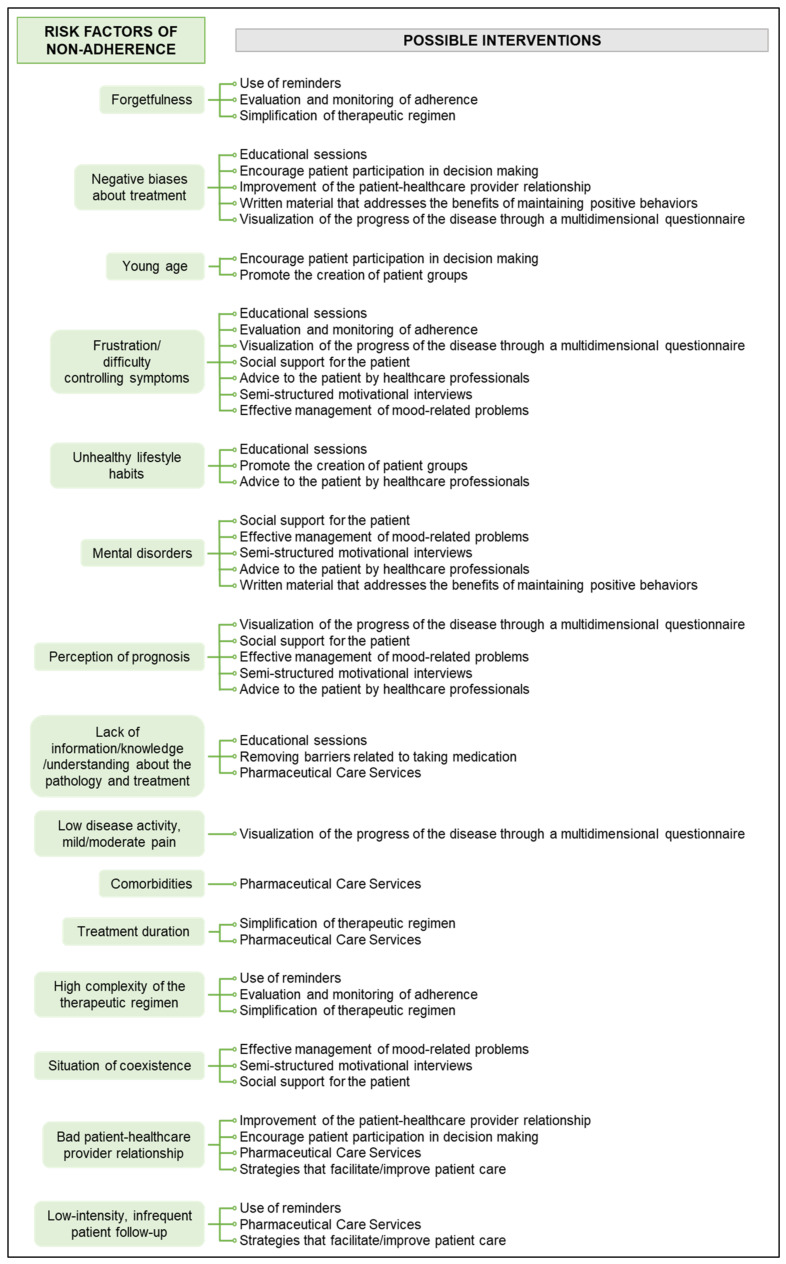
Map of risk factors and possible interventions to improve adherence.

Thus, each risk factor entails a series of possible specific interventions, as shown in the conceptual map in Figure 2.

## 4. Discussion

Therapeutic adherence depends on numerous factors, classified by the World Health Organization in five dimensions depending on their origin [1]: socioeconomic, healthcare system, patient, condition, and therapy. It should be noted that the WHO classification represents one perspective on adherence, but it may not be all inclusive. However, although this classification may seem limited, it includes a wide variety of risk factors for non-adherence, such as patients’ beliefs and perceptions about treatment or the severity of the disease, or the readability of directions of use. In this non-exhaustive review, many of them have been identified, but the interrelationships between all of them are complex and the divisions between dimensions are blurred in many practical cases.

Furthermore, some of the factors identified in the literature (such as patients’ treatment cost-related factors) cannot be extrapolated to any patient, disease, or healthcare system, and should therefore be assessed on an individual basis. For example, cardiac patients are particularly sensitive to the repercussions of non-adherence, since cardiovascular diseases have become the first cause of death worldwide and these patients need to take lifelong medications, often to treat silent conditions [156]. For this reason, awareness of the risk factors in this specialty area is key in optimizing patient outcomes.

Moreover, patients may vary their adherent behavior over time for various reasons, so adherence should be assessed on a regular basis. Ideally, non-adherence risk factors screening should be carried out prior to treatment initiation to predict the patient’s adherence, as well as a periodic follow-up of adherence. Patients’ individualized treatment and their personal experience with it should be evaluated to determine whether there is any factor that could lead to a lack of adherence. In fact, there are various strategies for predicting patients’ future adherence, including comprehensive initial follow-ups [157,158], as well as others based on machine learning [159,160]. Predicting, monitoring, and evaluating the factors associated with poor therapeutic adherence are the first steps to developing interventions that can counteract them.

To reach a consensus on shared non-adherence risk factors despite the existence of specific barriers to adherence related to specific diseases, the experts considered that some of the factors identified in the literature are common to different conditions and may represent a potential target for strategies to improve adherence in a more general way.

Implementing strategies to improve adherence should be a central part in health planning given how low adherence rates are associated with higher morbidity and mortality, as well as increased health costs. It should be noted that even the most effective, safe, and suitable treatment for any given patient becomes irrelevant if the therapeutic regimen is not followed as prescribed. In addition, adherence is a dynamic concept even within the same patient and condition, which highlights the importance of a proper follow-up. For these reasons, healthcare managers and professionals should be aware of the importance of identifying and assessing non-adherence risk factors, as well as developing and implementing interventions to achieve optimal patient adherence.

There are many ways to address non-adherence described in the literature. There are educational, behavioral, cognitive, affective, and multifaceted interventions for improving medication adherence, and it should be borne in mind that changing adherence patterns in patients with a long-term disease may be more difficult to achieve than developing new behaviors in newly diagnosed patients. This review proposes generic or multifaceted interventions that could improve adherence in patients with common non-adherence risk factors, which can be useful for clinicians and healthcare providers based on Steering Committee’s expertise. These interventions should be dynamic and customized to target populations or a person’s characteristics, in order to achieve and maintain better management of healthcare resources and improved patient health outcomes over time.

## 5. Conclusions

In this study, specific non-adherence risk factors in the fields of rheumatology, oncology, and cardiology are described, in addition to shared non-adherence risk factors, as well as a summary of interventions that can be implemented as potential strategies to improve adherence.

Despite the existence of specific factors for each pathology, it has been possible to identify common non-adherence risk factors between them and other chronic pathologies that have not been the subject of this study.

Taking this information into account, it is possible to identify key focus points to consider in the development of strategies to improve adherence. It is important to emphasize that the design of interventions must be adjusted to the specific characteristics of each patient and that the development of screening algorithms for adherence risk factors can be helpful in this regard.

Although no individual intervention with significant confidence in improving adherence in general has been identified, it is possible to design maps that interrelate risk factors and interventions or multifaceted interventions.

The Steering Committee has concluded that it may be interesting to develop screening tools for the identification of risk factors for non-adherence in patients, as well as for the proposal of interventions that could help achieve better health outcomes, in further projects. These tools could be very useful for healthcare providers.

## Figures and Tables

**Table 2:** Oncology risk factors of non-adherence.

Oncology	
**Socioeconomic**	Single marital status [54,55] Lack of social support [56,57,58,59,60,61,62] Living alone [63,64] High cost of medication [65,66]
**Healthcare** **system**	Bad patient–healthcare provider relationship [67,68] Short duration of care time per patient [56,69,70] Supply problems of community pharmacies [63] High cost of hospital care [71,72]
**Patient**	Young age (15–30 years) [54,73,74,75,76] Mental disorders [56,58,60,69,70,75,77,78,79,80,81,82,83,84] Negative biases about treatment [56,60,62,67,68,69,77,85,86,87,88] Negative biases about prognosis Lack of information and understanding about the disease and treatment [89,90,91] Frustration/difficulty of symptoms control [92] Forgetfulness [56,62,65,66,69,70,86,93,94,95,96,97,98] Unhealthy lifestyle habits [56,62,85,99]
**Condition**	Comorbidities [54,56,60,69,70,72,74,75,76,79,83,84,90,100,101,102,103,104,105,106,107,108,109] Perception of severity of disease [56,61,69,76,79,103,110,111] Perception of therapy’s risks and benefits [58]
**Therapy**	Adverse effects [56,62,70,80,91,94,96,98,105,106,112,113,114,115,116,117] High complexity of the therapeutic regimen [56,60,70,78,100,106] Treatment duration [56,60,70,96,98,115,118,119]

## Data Availability

Not applicable.
